# Perception of Stress and Styles of Coping with It in Parents Giving Kangaroo Mother Care to Their Children during Hospitalization in NICU

**DOI:** 10.3390/ijerph182312694

**Published:** 2021-12-02

**Authors:** Barbara Zych, Witold Błaż, Ewa Dmoch-Gajzlerska, Katarzyna Kanadys, Anna Lewandowska, Małgorzata Nagórska

**Affiliations:** 1 Institute of Health Sciences, Medical College of Rzeszow University, 35-310 Rzeszow, Poland; 2 Institute of Medical Sciences, Medical College of Rzeszow University, 35-310 Rzeszow, Poland; witekblaz@yahoo.com (W.B.); nagorska@ur.edu.pl (M.N.); 3 Department of Neonatology and Neonatal Intensive Care Unit, St Jadwiga Provincial Clinical Hospital No. 2 in Rzeszow, 35-301 Rzeszow, Poland; 4 Department of Didactics of Gynecology and Obstetrics, Medical University of Warsaw, 02-091 Warsaw, Poland; sekretariat.nzg@wum.edu.pl; 5 Department of Obstetrics, Gynecology and Obstetrics and Gynecology Nursing, Medical University of Lublin, 20-093 Lublin, Poland; katarzyna.kanadys@umlub.pl; 6 Institute of Healthcare, State School of Technology and Economics, Jaroslaw, 37-500 Jaroslaw, Poland; am.lewandowska@poczta.fm

**Keywords:** intensive care units, Kangaroo-Mother Care Method, neonatal, parents, stress

## Abstract

The experience of hospitalization of a newborn in the Neonatal Intensive Care Unit (NICU) may become distressing both for the baby and parent. The study aimed to assess the degree of parental stress and coping strategies in parents giving KMC to their babies hospitalized in NICU compared to the control group parents not giving KMC. The prospective observational study enrolled a cohort of 337 parents of premature babies hospitalized in NICU in 2016 in Eastern Poland. The Parental Stressor Scale: Neonatal Intensive Care Unit, Coping Inventory for Stressful Situations were used. The level of stress in parents giving KMC was defined as low or moderate. Analysis confirmed its greater presence in the group of parents initiating KMC late (2–3 weeks) compared to those starting this initiative in week 1 of a child’s life. An additional predictor of a higher level of stress in parents initiating KMC “late” was the hospital environment of a premature baby. Task oriented coping was the most common coping strategy in the study group. KMC and direct skin-to-skin contact of the parent with the baby was associated with a higher level of parental stress only initially and decreased with time and KMC frequency.

## 1. Introduction

### 1.1. Transfer—Placing the Newborn into the KC Position (Kangaroo Care)

Kangaroo-Mother Care Method (KMC) is the direct skin-to-skin contact of a parent with a baby. An undressed baby in a diaper (in the case of a premature baby dressed in socks and a cap) is placed on the naked chest of the parent. The child’s head is turned laterally to keep the airways open and allow eye contact with the parent, while the limbs are flexed along the trunk directly on the skin of the mother’s breast or father’s chest [1]. KMC should become a routine care method because of the undeniable evidence for its beneficial effects on the baby and parents. In addition, it is a “normal environment” that guarantees a continuous, almost 24-h skin to skin contact with frequent, exclusive or almost exclusive breastfeeding [2,3]. KMC is effective and simple tactile stimulation of the baby resembling natural care provided by the mother. It proved to be more beneficial than touching, stroking or even massage, therefore it is recommended to be started as soon as possible [2,4].

### 1.2. The Influence of KMC on the Behavior of the Newborn

Ensuring the baby’s sense of safety, KMC relieves negative emotions triggered by the environment, soothes and facilitates sleeping. Lower cortisol levels in the baby’s body resulting from stress reduction affects the immune system [5,6]. In addition, newborns given KMC cry less often and shorter than those remaining in the incubator [7]. In case of performing painful medical procedures, infants given KMC demonstrate significantly smaller pain response, less activity in response to a painful stimulus and, most importantly, significantly lower level of stress after surgery [3,8]. Therefore, KMC is recommended during stressful medical procedures (medical examination, nursing and therapeutic activities interrupting tissue continuity) [5,6]. Beneficial effect of KMC on the newborn was confirmed by randomized clinical trials and meta-analyses. Both heart rate and respiratory rate proved to be more stable during KMC than in an incubator. Changes in saturation level during KMC were minimal and within the normal range [1,8]. No drops in a newborn’s body temperature were observed, it remained normal or increased during KMC [1,9]. The colonization of the child’s skin with the physiological parental bacterial flora was also enhanced, reducing the prevalence of pre-existing risk of infection [10]. Infants during KMC were relaxed, calmer and fell asleep much easier with longer sleep. Sleep and awake phases were similar to those in full-term infants [11].

### 1.3. The Influence of KMC on the Development and the Psychosocial Sphere

KMC has a beneficial effect on breastfeeding. The number of breastfed infants on and after the discharge was higher in KMC group. KMC prolonged the breastfeeding period and increased lactation in mothers [4,12,13]. Better neurobehavioral development, eye-hand coordination, psycho-social reactions, hearing and speech were found in the KMC infant group [14]. Numerous studies proved that KMC strengthened parental feelings. Establishing a strong emotional bond with the baby during skin-to-skin contact enables both parents to adapt to a new situation and cope with the stress related to premature parenthood [14,15,16,17].

A preterm baby’s parents may experience constant fear of life and health of their offspring including hyperactivity of the HPA axis (hypothalamus-pituitary-adrenal) and uncertainty about its further development [8,18,19]. Literature review indicates the presence of many defined stressors resulting from the therapeutic process that implies neonatal and parental behavior in response to stress. Parental support from medical staff is becoming a key element in enhancing parent-baby interaction by developing strategies for preventive interventions in primary, secondary and tertiary care settings [20]. Undertaking KMC early in such cases helps to counteract the detrimental effects of separation for both the infant and the parent [14], but also to strengthen parental feelings related to childcare [12,19,21,22,23].

KMC reduces the feeling of parental anxiety, improves the parent’s self-esteem and acceptance of the child’s stay in the neonatal intensive care unit, also increasing his or her involvement in childcare [24,25]. It fosters a strong emotional bond with the child, parents feel responsible for the child, actively engage in care, feel more connected to the child and are more sensitive to the child’s needs [26].

Although beneficial impact of KMC has been recognized worldwide and is reflected in numerous studies, it is a relatively new form of care for the mother and the baby in Poland [27,28].

In our study, we examined a group of parents of premature babies born with moderately low body mass (1500–2499 g) and hospitalized in NICU. They constituted approximately 6% of all births in 2016 [29] of which 1.5% of parents were studied [30]. Literature review indicates the presence of many defined stressors resulting from the therapeutic process that implies neonatal and parental behavior in response to stress. Parental support from medical staff is becoming a key element in enhancing parent-baby interaction by developing strategies for preventive interventions in primary, secondary and tertiary care settings [20]. KMC continues to be a limited form of childcare for many parents in Poland, especially if it concerns sick and prematurely born children. Therefore, we decided to study the level of stress experienced by the parents giving KMC and their coping strategies. It is worth underlining that such studies have not been realized in Poland before.

### 1.4. Aim

The study aimed to assess the degree of parental stress and coping strategies in parents giving KMC to their babies hospitalized in NICU compared to the control group of parents not giving KMC.

A hypothesis was set that parents giving KMC to their children and starting KMC earlier experienced lower stress levels than parents not giving KMC and that most often they chose a Task-Oriented strategy for coping with stress.

## 2. Materials and Methods

### 2.1. Design

The prospective observational study was conducted among parents of preterm babies born in 2016 and hospitalized in NICU. The parents in the study were actively involved in caring for their own child (touch, feeding, routine care) and gave KMC, which is based on direct “skin-to-skin” contact. KMC time of the studied group was varied. For the purpose of the study, KMC was defined according to the criteria of KMC initiation and frequency.

### 2.2. Setting and Participants

The study group included 337 randomly chosen women and men, from the Eastern provinces of Poland whose children stayed in the NICU from January to December 2016 due to prematurity. All respondents were informed about the subject of the study, its purpose and the possibility of withdrawing from participation at any stage, and about anonymity.

Parents declaring their willingness to undertake KMC of their own child were informed about the survey and asked about their willingness to participate in it. Informed consent to participate in the study was confirmed with parent’s signature on the questionnaire form. Respondents participating in the study received a survey along with questionnaires in the envelope. During the study, the interviewer was present to answer the parents’ questions and doubts and collect completed questionnaires.

There were 39 parents who did not provide KMC for fear that this procedure combined with low birth weight could negatively affect their offspring’s health status. 

### 2.3. Data Collection

The research tool used was the information survey developed by the authors, the Polish version of Parental Stressor Scale: Neonatal Intensive Care Unit (PSS-NICU) [31] and Coping Inventory for Stressful Situations (CISS) [32].

The information survey was developed solely for the purpose of this study and served to collect parents’ and their children’s sociodemographic and medical data. The respondents were asked about sex, age, education and clinical data on the course of the current pregnancy (diseases coexisting with pregnancy, ailments resulting from diseases coexisting with pregnancy, hospitalizations during pregnancy and type of birth). The child-related questionnaire contained questions about sex, type of pregnancy according to the number of babies born. The body weight, length at birth and general condition on the Apgar scale at 1, 3, 5 and 10 min of the child’s life were collected from the medical records by a group of midwives trained for this purpose. The information survey regarded parents’ sex, age, education and infant’s sex, weight and health at birth, initiation, frequency and duration of KMC. Information on the birth weight and Apgar scores were additionally confirmed by the analysis of the medical documentation of the newborn.

PSS-NICU includes three scales of stress generated by hospital environment: sights and sounds related to therapeutic and diagnostic processes (subscale I), infant appearance and behavior (subscale II) and parental role alternation during hospitalization and treatment of the baby, the equipment used and the relationship between parents and the staff (subscale III). The survey includes a total of 34 questions. The degree of stress in subjects was determined in a 5-point scale (from 1-Not at all stressful to 5-Extremely stressful). In the case of the situation described as neutral or not experienced during child’s hospitalization in NICU, the answer was scored 0 pts. The tool was originally developed by Miles et al. [31]. The present study used the Polish version of the above-mentioned tool.

After obtaining the permission of the author of the PSS-NICU Questionnaire, the original version of the research tool was translated into Polish by two independent English translators whose native language was Polish. Both translators were graduates of English Philology and they were professionally involved in translating and teaching English at university. During the preparation of the Polish version of the questionnaire, the identical graphic form of the questionnaire was used, and the selection of the study group and data collection was in line with the recommendations of the PSS-NICU questionnaire. The internal compliance index α-Cronbach for the PSS questionnaire: NICU adopted for a group of KMC parents of their children in the ICU was 0.94. The reliability of individual subscales turned out to be equally high, for subscales I: 0.75, for subscales II: 0.92 and for subscales III: 0.86.

At the time when we were implementing our project, the PSS: NICU tool was also independently adapted by two Polish teams: Libera [33] and Aftaka [34].

Libera A. et al. dealt with the adaptation of the scale by M.S. Miles in Poland in 2013 to determine basic psychometric characteristics (validity and reliability) of the Polish version of PSS: NICU for 3 subscales: clinical status of the child and medical procedures, personal and interpersonal problems, and maternal competences [33]. The internal compliance index calculated for α-Cronbach is 0.96 in the range from 0.34 to 0.69. The correlation half-factor between subscales ranged from 0.46 to 0.63 and proved to be accurate and reliable in studies on the level and structure of stress experienced by women in prematurity [33]. Aftaka A et al. conducted further studies in 2019 on the Polish adaptation of PSS: NICU was high. For the entire scale, Cronbach’s alpha was 0.92, for the Infant Appearance subscale: 0.92, for the Parental Role Alteration sub-scale: 0.86, and for the Sights and Sounds sub-scale: 0.78. The values of Cronbach’s alpha allow for the application of the tool in empirical research and individual diagnosis [34].

The questionnaire for Coping in Stressful Situations (CISS), developed by Endler and Parker [32,35], adapted to Polish conditions by Strelau J, Jaworska A, Wrześniowski K (2009) was also used [36]. CISS assesses stress coping strategies. It consists of 48 items evaluated in a five-point Likert scale: 1-never, 2-very rarely, 3-sometimes, 4-often, 5-very often. It measures three styles of coping with stress: Task-Oriented Coping (TOC), Emotion-Oriented Coping (EOC) and Avoidance-Oriented Coping (AOC) in two subscales: engaging in substitute activities (subscale ST) and seeking social contacts (subscale SC). Each style includes 16 items, which means that the range of results for each style is from 16 to 80 points, for ST the range is 8–40 points, for SC—5–25 points. The higher the score for a given style, the more often the person uses the strategies included in it. CISS has been normalized in the Polish adaptation (2003) using STEN norms for three age groups (16–24, 25–54, 55–79), where the value 1–4 STEN corresponds to a low score, 5–6 STEN—an average score, and 7–10 STEN—the high score [36].

In terms of reliability measured by the α-Cronbach coefficient, the data obtained from the study of three groups are very consistent. Within the individual scales, the coefficients differ by 0.02–0.06, assuming values for the SSZ and SSE scales in the range from 0.82 to 0.88, and for the SSU scale from 0.74 to 0.78. In addition to the PKT subscale, which in two groups of respondents showed reliability below the critical value of 0.70, the reliability of the scale is considered satisfactory by the authors [36], despite the fact that it is slightly lower (from 0.02 to 0.14, on average by 0.05) than in the case of the scale of the original version [36].

The internal coherence and reliability of the CISS questionnaire, as measured by the α-Cronbach coefficient in the area of stress management styles of parents of children in the ICU, was at a high level. For all the parents surveyed, it was 0.95. For a task-oriented style (TOC: 0.87), an emotion-oriented style (EOC: 0.89) and avoidance style (AOC: 0.83) in two subscales: engaging in surrogate activities (ST subscale: 0.81) and seeking social contacts (subscale S.C.: 0.75) should be considered satisfactory.

### 2.4. Measurements

For the purpose of the study, KMC was defined according to three criteria: the first criterion was parents giving KMC vs. not giving KMC; the second—KMC initiation (week 1 of the baby’s life vs. week 2 of the baby’s life or later); the third criterion—KMC frequency (KMC daily vs. KMC intermittently).

1. Parents giving KMC vs. not giving KMC: the subjects giving KMC were a group who had the opportunity to hold the child in the diaper (in socks and cap), lying with arms and legs folded to their own torso, and the belly adhered directly to the naked skin between the mother’s breasts or the father’s torso. The parents not giving KMC were a group who did not participate in this kind of initiative or were a passive observer of KMC of their child. The criteria for including a parent in the study were the newborn’s kangaroo care and informed consent to participate in the study. The criterion for enrolling the newborn in the study was 27 weeks of age; stable clinical condition; independent breathing or breathing with a respirator; trophic, enteric or parenteral feeding; not feeling intense pain and without the presence of a catheter in the umbilical vessels.

The criterion for exclusion from the study was the lack of parental consent to participate in the study. On the part of the newborn, unstable general condition, i.e., suspected infection or sepsis; sensation of intense pain, early postoperative period; situations requiring medical intervention (hernia, eberitude), necrotizing enterocolitis, drainage of the pleural cavity and rapidly increasing levels of bilirubin despite the phototherapy used.

2. Parents giving KMC from week 1 of child’s life vs. week 2 of the child’s life. Parents giving KMC to their children in the first week of the child’s life constituted a group beginning KMC from the 1st to the 7th day of the child’s life. Parents giving KMC to their child from the 2nd week of the child’s life implemented it between the 8th and 21st day of a child’s life. Stable clinical condition of the child and the parent’s readiness to start and continue KMC were conditions allowing for KMC initiation.

3. KMC daily vs. KMC intermittently. KMC daily included parents performing KMC for at least one hour per day. Parents giving KMC to their children intermittently constituted a group performing KMC on average 4 times a week.

### 2.5. Data Analysis

Statistical analysis was performed with Statistica 10.0 PL, the database and charts were developed in Microsoft Excel. The analysis of the variables was performed with non-parametric tests verified by the Shapiro-Wilk test. The differences in average numerical properties in two populations were evaluated with Mann-Whitney U test. The correlation of two numerical variables that did not meet the criteria of normality was calculated using Spearman’s rank correlation coefficient. To assess the reliability coefficient of the PSS: NICU and CISS questionnaires, the Alpha Cronbach coefficient was calculated. The level of significance was adopted at *p* < 0.05.

### 2.6. Ethical Statement

The study was conducted according to the guidelines of the Declaration of Helsinki and approved by the Institutional Review Board at the University of Rzeszow (protocol code No. 12/02/2013 date 27 February 2013).

## 3. Results

In total 337 parents of premature infants were included in the study, the majority of them were women. The mean age of the surveyed parents was 30 (30.66 years ± 5.16), the number of infants was 398, the vast majority were single pregnancies, moderately low birth weight and of average health condition. Detailed sociodemographic and clinical data of the studied group of parents and children are presented in Table 1.

### 3.1. The Stress Parents Giving KMC to Their Children during Hospitalization in NICU

The factors causing the highest intensity of stress in all surveyed parents of the infants hospitalized in NICU were the items related to the infant’s appearance and behavior (group II) and parental role alternation related to hospitalization and treatment of the baby (group III). The highest level of parental stress was generated by baby’s unusual or abnormal breathing patterns (4.59 pts.), baby’s suffering (4.5 pts.), feeling helpless and unable to protect the baby from pain and painful procedures (4.33 pts.), feeling helpless about how to help the baby during this time (4.32 pts.), and the limp and weak appearance of the baby (4.25 pts.).

**Figure 1:**
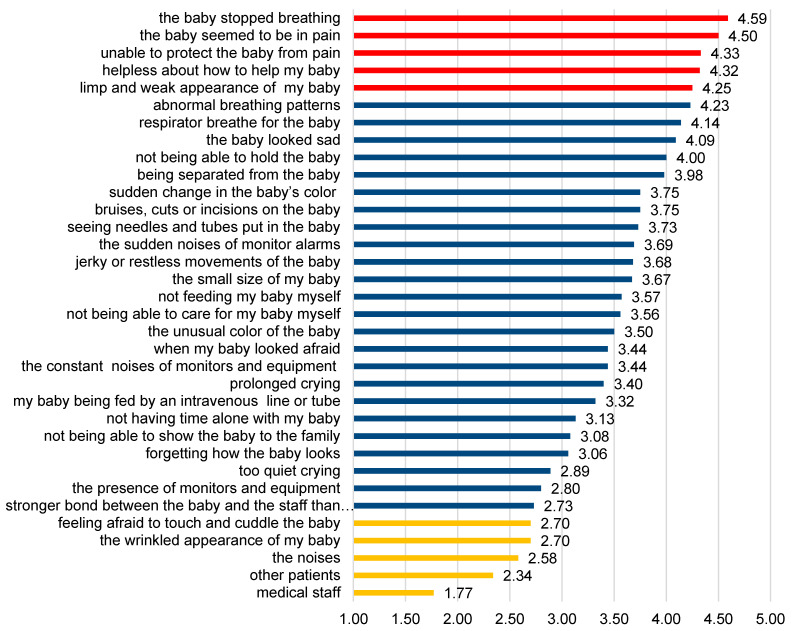
Summary of the results of the PSS-NICU questionnaire among all surveyed parents. Legend: PSS-NICU three scales of stress generated by hospital environment: Red: sights and sounds related to therapeutic and diagnostic processes (subscale I). Blue: infant appearance and behavior (subscale II). Yellow: parental role alternation during hospitalization and treatment of the baby, the equipment used and the relationship between parents and the staff (subscale III).

The least stress was generated by the therapeutic and diagnostic process and concerned the presence of medical staff (1.77 pts.), other sick babies in the room (2.34 pts.). Graphical data of the sources of stress in the hospital environment among the parents surveyed is shown in Figure 1.

The three-component version of the PSS-NICU questionnaire showed a significant difference in the therapeutic and diagnostic process (subscale I) in the group of parents giving KMC, compared with parents not giving KMC (criterion I: *p* = 0.006), in whom higher stress levels were noted (2.85 ± 0, 86 vs. 2.45 ± 0.94). In other categories of stress sources (subscale II, subscale III) no significant differences were observed in the criterion of KMC initiation (criterion II: 2.75 ± 0.89 vs. 2.98 ± 0.74) and its frequency (criterion III: 2.84 ± 0.86 vs. 2.84 ± 0.85), although the result of the therapeutic and diagnostic process (subscale I) was close to *p* = 0.05 for parents starting KMC from the second week of the child’s life. In other cases, the results of the PSS-NICU category were not statistically significant.

A general analysis of parental stress confirmed the significant difference in the group of parents beginning KMC of children from the second week of life in comparison with parents initiating it in the first days of the child’s life (1st week of the child’s life). In the remaining cases, the level of parental stress did not show significant differences between particular groups of measured variables (Table 2).

### 3.2. Coping Strategies in Parents Giving Kangaroo Mother Care to Their Children during Hospitalization in NICU

In the group of parents surveyed, Task-Oriented Coping (TOC) was the most frequent strategy for coping with stress. Comparable results were obtained among parents giving KMC and not giving KMC who showed their tendency to solve problems through a task or planning. Definitely this strategy was slightly more frequent in the group of parents giving KMC to their children daily from week 1 of the child’s life. The obtained mean in this group corresponds to the 6th STEN according to Polish standards (Table 3 and Table 4).

Emotion-Oriented Coping (EOC: 5 STEN according to Polish standards) was the next strategy to cope with stress in parents giving KMC to their children intermittently, but from week 1 of their life. In this case, parents showed a tendency towards wishful thinking and focused on their own experiences and emotions (Table 3 and Table 4).

The least frequently expressed strategy of coping with stress by parents was Avoidance oriented coping (AOC) (4 STEN according to Polish standards) despite the fact that the results show only a higher intensity among parents giving KMC to their children from birth but intermittently. This style is presented in two forms: engaging in substitute activities (ST) (5 STEN according to Polish standards), or seeking social contacts (SC) (4 STEN according to Polish standards). ST was more frequent in AOC in the parents giving KMC, than SC, which was indicated by parents who gave KMC intermittently from week 1. Only a higher intensity of statistical trend can be observed in each case (Table 4).

## 4. Discussion

Our research confirmed the parents’ feelings of strong stress related to the hospital environment and the therapeutic and diagnostic process of the child in NICU. It turned out that high intensity of stressful factors at such level compared to parents not giving KMC were observed only at the beginning of parental intervention. Despite the fact that we did not confirm the influence of three sources of stress in the assessment of general parental stress, we observed it at a low (“2”) or moderate (“3”) level. Our results are supported by the findings of other researchers [19,37,38]. They indicate the birth of a premature baby as a stressful experience for the parent, but the level of stress assessed was moderate. Among the stressors of the therapeutic and diagnostic process of the NICU environment, the strongest effects on the parents were images (appearance and behavior of the child) and sounds [17,29,39,40,41].

In our study we also noticed that the stress of parents giving KMC to their children from the first hours of their life was close to statistical significance in terms of diagnostic and therapeutic process, compared with parents starting this initiative from week 2 of the child’s life or later. In the assessment of total parental stress, a longer time of parental intervention influenced the reduction of stress. We suppose that, in this particular situation, the appearance and behavior of a preterm infant characteristic of its gestational age could have a direct impact on the time of initiation of parental intervention, and was indirectly associated with a change in parental role alternations. Our results are also in line with the study by Bouet K.M. [42] which indicate that the highest level of stress experienced by parents of premature babies concerned the parental role alternations as well as the appearance and behavior of the child. However, in order to reduce the level of stress, Dudek-Shriber L. claims that, emphasis should be placed on family-centered care [43].

The results of our study among parents giving KMC are confirmed by observations of other authors [14,28]. An effective intervention reducing maternal stress is described by Kadivar et al., who point to the fact that parental stress decreases between day 3 and 10 from the beginning of the intervention, up to the first half of the child’s life, due to better social and neurodevelopmental development of infants [20].

However in order for this kind of parental care to be started as soon as possible, the medical staff must become the “intermediary” of parents to teach them to properly interpret the child’s signals and engage them during care [44]. Then coping with stress becomes a parent’s disposition to respond to a stressful situation [45,46] and the functioning of the family [18,47]. Undertaking resolute remedial actions, by regulating emotions and constructively solving problems, contributes to self-protection against stress [48]. Nowadays the predominant approach to the phenomenon of stress is transactional. The nature of a given stressful situation stimulates human activity, to regain a balance between the requirements and the possibilities to improve the emotional state [46].

In the sense of stress as a transaction, disruption of the balance between the requirements and the possibilities of their fulfillment, the stressful situation will be the care of a premature baby in ICU. Remedial actions taken by staff in a given stressful situation are the result of interaction between the characteristics of the situation and the individual style of coping [49,50]. TOC in our case is one of effective styles of coping with stress in parents giving KMC and confronting the problem. To a lesser extent, this style is used among parents not giving KMC. Similar observations were made in the group of women giving premature birth [48] by K. Mariańczyk et al. who indicated that TOC is used to a lesser extent by women after preterm delivery compared to women after full-term childbirth. On the other hand, in terms of EOC and AOC, no significant differences are noticed between the studied groups [48].

In our study, the second style of coping with stress in KMC parents and non KMC parents is EOC. The level of concentration on oneself and negative experiences in this group of parents corresponds to the mean values, and the obtained results lead to the belief that mothers and fathers similarly experience parenthood regardless of initiation and frequency of skin-to-skin contact.

Similar results were obtained by Ghorbani et al. in the studies of the parents of premature and full-term babies [51]. Their results show no significant difference between the two groups of mothers and fathers in terms of coping strategies. Mothers of premature babies slightly more often responded with EOC or AOC, while fathers used every stress management style compared to those of the full-time babies’ parents [51]. EOC was also observed in the group of mothers of children with physical disabilities and was associated with their lower self-esteem compared to the mothers of children with normal development [37]. Despite the emerging negative evidence of the occurrence of possible adaptation difficulties among people using EOC [52] and described difficulties in coping with stress in this particular group [46,53]. Based on our study, we believe that EOC can be an adaptive potential among our parents due to their regulatory function.

In our study, we have also noticed AOC was the least popular. This form of coping was chosen by parents giving KMC to their babies from birth intermittently, compared to parents who gave KMC to their babies from week 2 daily. According to Cattell, people presenting AOC displace from their awareness stressful situations [54] which they have no influence on. This situation is particularly strongly experienced among families of children with dysfunctions requiring constant respiratory support [55]. In our opinion, this situation may indicate that parents confront trauma of premature birth and prematurity not addressed mentally yet and the various health problems of the child appearing at that time.

Regardless of the frequency of interventions involved in KMC, the parents studied by us showed a higher level of TOC, although it remains within the average limits for the Polish population. Such diverse ways of coping with stress among parents may indicate their better mental condition, related to professional care provided to their children and receiving integrated support, counteracting stress experienced in NICU [52,56].

Our study had also some limitations. Although more children were born in Poland in the last two years. In 2016, the number of viable births amounted to 382,000 and was higher by 13,000 than in the previous year, and in 2017, approx. 402,000 children, i.e., another 20,000 more. The number of children born with low body weight (less than 2500 g) during our study was less than 6% (5.7% in 2016) in relation to the total number of deliveries [30] of which we surveyed 1.5% of parents. The limited number of parents examined by us was related to the clinical condition of their children and the length of their stay in the ward, and it results from health problems that appear the more often, the more immature premature baby is [57]. We are aware that the research results obtained by us represent only a small part of KMC’s impact on the obtained results of the study are only a part of the impact of KMC on the level of stress experienced by parents and ways to counteract it, however, they require future multicenter randomized trials confirming the results of our research.

## 5. Conclusions

Undertaking KMC related to the direct contact of naked skin of a parent with a child, showed a higher level of parental stress only in the initial period of hospitalization and treatment of the child. Moreover, delaying the time of KMC initiation (between week 2 and 3) implicated a higher level of parental stress compared to the caregivers starting the initiative from the birth of the child. An additional predictor of the higher level of stress among parents starting “late” KMC is the hospital environment of the premature baby.

Among parents, the dominant strategy for coping with stress was task-oriented coping. In spite of the lack of significant differences in coping with the stress in parents giving KMC to the preterm, it certainly is a big contribution in the process of adaptation to the role of a child’s career. We believe that this research can be considered innovative, and the method of recommended parental care in Poland may affect our thinking, how to care for parents, not only focusing on the physical consequences of separation with the child.

## Figures and Tables

**Table 1:** Sociodemographic and clinical data of the studied group of parents and children.

**Sociodemographic Data of the Tested Parents**		**n, (%)**
Gender	Female	261, (77.4)
	Male	76, (22.6)
Age	≤24 yrs	38, (11.3)
	25–34 yrs	232, (68.8)
	≥35 yrs	67, (19.9)
Education	Secondary education	147, (43.6)
	Higher	190, (56.4)
**Clinical Data of the Course of the Present Pregnancy**		**n, (%)**
Conditions coexisting with the pregnancy	Yes	96, (28.5)
	No	241, (71.5)
Ailments resulting from conditions coexisting with the pregnancy	Yes	130, (38.6)
	No	207, (61.4)
Hospitalizations in pregnancy	Yes	227, (67.4)
	No	110, (32.6)
Number of hospitalizations in pregnancy	One	123, (54.2)
	Two	67, (29.5)
	Three or more	37, (16.3)
Delivery method	Vaginal birth	59, (17.1)
	Caesarean section	278, (82.9)
**Sociodemographic and Clinical Data of the Infant**		
Infant’s gender	Female	217, (54.5)
	Male	181, (45.5)
Single vs. Multiple Pregnancy	Single Pregnancy	279, (82.8)
	Multiple Pregnancy: twins	58, (17.2)
Week of pregnancy completion	<29	107, (26.9)
	29–32	161, (40.4)
	33–36	111, (27.9)
	>36	19, (4.8)
Body weight at birth	<1000 g	75, (18.8)
	1000–1499 g	93, (23.4)
	1500–2499 g	161, (40.4)
	≥2500 g	69, (17.4)
Body length n, (x ± SD)	23–63 cm	398, (44.00 ± 7.88 cm)
Apgar score n, (x ± SD)	1st minute	304, (5.71 ± 3.16 pts)
	3rd minute	188, (5.21 ± 3.27 pts)
	5th minute	217, (6.01 ± 3.31 pts)
	10th minute	203, (6.28 ± 3.49 pts)
**Data on Undertaking KMC in Parents**		**n, (%)**
Undertaking KMC	Yes	298, (88.4)
	No	39, (11.6)
KMC Initiation	Week 1 of infant’s life	194, (65.1)
	Week 2–3 of infant’s life	104, (34.9)
KMC Frequency	Daily	167, (56.0)
	Intermittently	131, (44.0)

**Table 2:** List of results of the PSS-NICU questionnaire among the surveyed parents, taking into account undertaking KMC in the hospital, initiation and frequency.

**PSS-NICU and KMC**	**Parents Giving KMC**			**Parents Not Giving KMC**			***p***
	$\bar{x}$	**Me**	**SD**	$\bar{x}$	**Me**	**SD**	
Subscale I	2.85	2.83	0.86	2.45	2.42	0.94	0.006 *
Subscale II	3.67	3.82	0.89	3.38	3.38	1.01	0.093
Subscale III	3.58	3.70	0.95	3.33	3.27	1.08	0.198
The general level of stress in parents:	3.35	3.39	0.77	3.07	2.97	0.91	0.056
**PSS-NICU and KMC Initiation**	**KMC from Week 1 of Infant’s Life**			**KMC from Week 2 of Infant’s Life**			***p***
	$\bar{x}$	**Me**	**SD**	$\bar{x}$	**Me**	**SD**	
Subscale I	2.75	2.80	0.89	2.98	3.00	0.74	0.050
Subscale II	3.59	3.76	0.95	3.75	3.88	0.78	0.254
Subscale III	3.49	3.44	1.00	3.72	3.88	0.82	0.058
The general level of stress in parents:	3.26	3.31	0.80	3.48	3.50	0.66	0.022 *
**PSS-NICU and KMC Frequency**	**KMC Daily**			**KMC Intermittently**			***p***
	$\bar{x}$	**Me**	**SD**	$\bar{x}$	**Me**	**SD**	
Subscale I	2.84	2.83	0.86	2.84	2.83	0.85	0.975
Subscale II	3.68	3.82	0.87	3.64	3.75	0.90	0.653
Subscale III	3.57	3.67	0.94	3.60	3.73	0.95	0.781
The general level of stress in parents:	3.35	3.36	0.76	3.34	3.42	0.76	0.908

$\bar{x}$—arithmetic mean; Me-median; SD—standard deviation; *p*—level of probability for the Mann-Whitney U test; * *p* < 0.05.

**Table 3:** Evaluation of the stress coping strategy for the overall number of parents surveyed.

Evaluation of the Stress Coping Strategy	Low 1–4 STEN		Average 5–6 STEN		High 7–10 STEN	
	n	%	n	%	n	%
Task-Oriented Coping	63	18.7	129	38.3	145	43.0
Emotion-Oriented Coping	103	30.6	217	64.4	17	5.0
Avoidance-Oriented Coping	208	61.7	89	26.4	40	11.9
search for social contacts	171	50.7	101	30.0	65	19.3
engaging in substitute activities	91	27.0	155	46.0	91	27.0

**Table 4:** List of CISS scale results among respondents with respect to the fact of undertaking KMC, its initiation and frequency.

**CISS and KMC**	**Parents Giving KMC**			**Parents Not Giving KMC**			***p***
	$\bar{x}$	**Me**	**SD**	$\bar{x}$	**Me**	**SD**	
Task-Oriented Coping	59.78	60.00	8.77	60.77	62.00	9.49	0.340
Emotion-Oriented Coping	42.30	42.50	10.10	41.87	42.00	12.12	0.721
Avoidance-Oriented Coping	38.03	37.50	8.04	36.92	35.00	8.55	0.218
search for social contacts	16.34	16.00	5.57	15.36	14.00	5.71	0.188
engaging in substitute activities	16.64	16.00	3.46	16.18	17.00	3.36	0.496
**CISS and KMC Initiation**	**KMC from Week 1 of Baby’s Life**			**KMC from Week 2 of Baby’s Life**			***p***
	$\bar{x}$	**Me**	**SD**	$\bar{x}$	**Me**	**SD**	
Task-Oriented Coping	59.90	60.00	8.86	59.59	60.00	8.61	0.913
Emotion-Oriented Coping	42.18	42.00	10.75	41.97	44.00	9.30	0.985
Avoidance-Oriented Coping	38.48	38.00	8.45	36.78	36.00	7.40	0.101
search for social contacts	16.74	16.00	5.83	15.36	15.00	5.12	0.065
engaging in substitute activities	16.71	17.00	3.61	16.27	16.00	3.27	0.150
**CISS and KMC Frequency**	**KMC Every Day**			**KMC Intermittently**			***p***
	$\bar{x}$	**Me**	**SD**	$\bar{x}$	**Me**	**SD**	
Task-Oriented Coping	60.08	60.00	8.95	59.71	60.00	8.56	0.807
Emotion-Oriented Coping	41.99	42.00	10.54	42.47	43.00	9.38	0.545
Avoidance-Oriented Coping	37.54	37.00	7.87	38.75	38.00	8.44	0.219
seeking social contacts	15.98	16.00	5.54	16.83	16.00	5.69	0.246
engaging in substitute activities	16.57	16.00	3.57	16.72	17.00	3.41	0.677

$\bar{x}$—arithmetic mean; Me-median; SD-standard deviation; *p*—level of probability for the Mann-Whitney U test; * *p* < 0.05.

## Data Availability

The data analyzed in the study are available upon request to the corresponding author.
